# The Effect of Comfort Care on Postoperative Quality of Life, Psychological Status, and Satisfaction of Pancreatic Cancer Patients

**DOI:** 10.1155/2022/9483762

**Published:** 2022-05-30

**Authors:** Yingliang Miao

**Affiliations:** Department of Gastroenterology, Hai'an People's Hospital, Nantong, China

## Abstract

**Objective:**

To evaluate the effect of comfort care on postoperative quality of life, psychological status, and satisfaction of pancreatic cancer patients.

**Methods:**

From June 2019 to March 2021, 136 pancreatic cancer patients undergoing pancreatectomy in Hai'an People's Hospital were recruited and randomly assigned via the random number table method at a ratio of 1 : 1 to receive either conventional care (control group) or comfort care (study group), with 68 cases in each group.

**Results:**

Before the intervention, the two groups had similar visual analog scale (VAS) scores, the Medical Outcomes Study 36-Item Short-Form Health Survey (SF-36) scores, and psychological status scores. The study group resulted in a significantly lower VAS score than the control group. The study group required a lower dose of analgesics than the control group. After the intervention, the study group showed significantly higher scores in social functioning, role emotional, mental health, role physical, and bodily pain than the control group. The study group had significantly lower Self-Rating Anxiety Scale (SAS) and Self-Rating Depression Scale (SDS) scores than the control group. The study group showed a significantly lower incidence of complications and a higher satisfaction rate than the control group.

**Conclusion:**

Comfort care effectively alleviates the pain of patients after pancreatectomy, reduces the incidence of complications, and improves their quality of life, psychological status, and satisfaction, so it is worthy of clinical application.

## 1. Introduction

Pancreatic cancer is a common tumor of the gastrointestinal tract with a high degree of malignancy [1, 2]. Its clinical manifestations vary depending on the lesion site, disease course, metastasis, and adjacent organ involvement, and common symptoms include upper abdominal discomfort or abdominal pain, abdominal mass and ascites, jaundice, and weakness [3]. With insidious early symptoms, rapid progression, high surgical mortality, low cure rate, and poor survival, pancreatic cancer is considered one of the malignant tumors with the worst prognosis. According to the Lancet, the five-year survival of pancreatic cancer after diagnosis is only about 10% [4]. Moreover, a report from the World Health Organization (WHO) in 2012 showed that the global prevalence and mortality rate of pancreatic cancer ranked 13th and 7th in malignant tumors, respectively [5], and data from the National Cancer Center of China showed that the incidence and mortality rate of pancreatic cancer ranked 10th and 6th in malignant tumors in 2019, respectively, with a male-to-female ratio of 1.5–2 : 1 [6]. Currently, surgical resection is the clinical radical treatment for pancreatic cancer. However, a large body of evidence shows a poor surgical outcome and unfavorable five-year postoperative survival.

It has been confirmed that surgical outcomes could be ameliorated by efficient postoperative care interventions. Comfort care [7] is a new and effective nursing modality providing physiological and psychological care to enhance patient comfort and satisfaction [8, 9]. It includes care in the postural comfort to prevent compression of nerves and blood vessels, environmental comfort to help the patient maintain a positive treatment attitude, temperature and humidity management to ensure physical comfort [10], and active communication and psychological guidance to eliminate negative emotions [11]. Different from the traditional nursing concept, comfort care focuses on the mitigation of patients' unpleasantness and psychological disorders management of patients in treatment. However, the nursing efficiency of comfort care for postpancreatectomy patients is marginally explored. Accordingly, this study was undertaken to evaluate the effect of comfort care on postoperative quality of life, psychological status, and satisfaction of pancreatic cancer patients to provide a reference for the clinical postoperative care of pancreatic cancer patients.

## 2. Materials and Methods

### 2.1. Participants

Between June 2019 and March 2021, 136 patients with pancreatic cancer (77 males and 59 females, aged 26–71 years, mean age of 42.89 ± 5.08 years) undergoing pancreatectomy in our hospital were recruited and assigned via the random number table method at a ratio of 1 : 1 to receive either conventional care (control group) or comfort care (study group), with 68 patients in each group The research was approved by the Ethics Committee of Hai'an People's Hospital (97770/1)

### 2.2. Inclusion and Exclusion Criteria

Patients who met the diagnostic criteria for pancreatic cancer after examinations; who underwent pancreatectomy in our hospital; and who provided written informed consent were included in the present study.

Patients with serious dysfunction of the heart, liver, and kidney; with psychiatric diseases or unconsciousness; and who revoked their consent were excluded.

### 2.3. Treatment

Patients in the control group received conventional care, including monitoring of vital signs, instructions on antibiotics use, hemostatic and analgesic drugs administration, observation of the properties and color of drains, and timely management of other medical conditions such as hemorrhage. One day postoperatively, nursing staff helped patients perform out-of-bed activities and provided discharge instructions and postdischarge follow-up.

Patients in the study group received comfort care. (1) Environmental care: the ward was maintained quiet, and the temperature, humidity, and light in the ward were regulated to provide the patients with a good recovery environment. (2) Psychological care: patients may experience psychological pressure and anxiety after surgery due to postoperative pain and fear of disease recurrence, so the psychological changes in patients were closely monitored. Targeted psychological care protocols were developed according to each patient's situation for the management of psychological disorders of patients. (3) Social comfort care: the nursing staff actively communicated with the patient's family members and timely informed them of the patient's physical and psychological condition, which contributed to potentiating the psychological care efficiency and helped the patients strengthen their treatment confidence. (4) Postoperative comfort care: patients with severe pain were given intramuscular injections of analgesics intermittently, and analgesic pumps were used when necessary. The patients were also given dietary guidance and were advised to follow a high-calorie, high-protein diet, low-fat, and low-salt diet after surgery to ensure a balanced intake of nutrients. The nursing staff closely monitored the changes in patients' vital signs and drainage fluid and timely informed the doctors of any abnormalities, so as to maximize the physical and mental comfort of patients after surgery.

### 2.4. Outcome Measures

1. Patients' pain level was rated using the visual analog scale (VAS) with a score of 0–10 points [12]. A score of ≤3 points indicated mild pain with no effect on daily life, 4–6 points indicated moderate pain that was tolerable, and ≥7 points indicated severe pain that was unbearable. The scores were proportional to the degree of pain, and the use of analgesics in the two groups was recorded in detail and compared.
2. The patients' quality of life was assessed using the MOS 36-Item Short-Form Health Survey (SF-36) [13], which was divided into physical health (physical functioning, role physical, bodily pain, general health) and psychological health (vitality, social functioning, role emotional, mental health). Each domain had a total score of 100 points, and higher scores indicated better quality of life of patients.
3. The patients' negative emotions were assessed using the Self-Rating Anxiety Scale (SAS) and the Self-Rating Depression Scale (SDS) [14], both with a total score of 100 points. For SAS, a score of 50–70 points indicated mild anxiety, 71–90 points indicated moderate anxiety, and >90 points indicated severe anxiety. For SDS, a score of 53–62 points indicated mild depression, 63–72 points indicated moderate depression, and 72 points or more indicated severe depression. The higher the score, the more severe the patient's anxiety and depression.
4. The complications of the two groups were recorded in detail, including infection, pressure sores, oral ulcers, and venous thrombosis. The total incidence of complications in the two groups was calculated and compared.
5. The “Nursing Satisfaction Questionnaire” was adopted for nursing satisfaction evaluation including three items, namely, the attitude of medical staff, nursing efficiency, and disease education. The questionnaire was designed by our hospital, and the result was divided into four levels (highly satisfied, satisfied, less satisfied, and dissatisfied) to obtain the satisfaction of patients in both groups with Cronbach's *α* of 0.921.

### 2.5. Statistical Analysis

GraphPad Prism 8 software was used to plot the graphics, and SPSS22.0 software was used for data analyses. Count data are expressed as *n* (%) and analyzed using the chi-square test, and measurement data are expressed as mean ± standard deviation and analyzed using Student's *t*-test. Differences were considered statistically significant at *P* < 0.05.

## 3. Results

### 3.1. Baseline Patient Profile

The baseline characteristics of the control group (38 males and 30 females, aged 26–71 years, mean age of 43.03 ± 4.87 years, disease duration of 1–4 years, mean disease duration of 2.23 ± 0.71 years, 24 cases of highly differentiated adenocarcinoma, 25 cases of moderately differentiated adenocarcinoma, and 19 cases of ductal cell carcinoma) were comparable with those of the study group (39 males and 29 females, aged 28–70 years, mean age of 42.65 ± 5.61 years, disease duration of 1–4 years, mean disease duration of 2.37 ± 0.58 years, 26 cases of highly differentiated adenocarcinoma, 24 cases of moderately differentiated adenocarcinoma, and 18 cases of ductal cell carcinoma) (*P* > 0.05) (Table 1).

### 3.2. VAS Scores and Analgesic Use

Before the intervention, the two groups had similar VAS scores (*P* > 0.05). After intervention, the study group had a significantly lower VAS score (2.17 ± 0.31) than the control group (6.51 ± 0.98) (*P* < 0.05) (Table 2). The medication rate was 70.59% (48/68) in the control group and 35.29% (24/68) in the study group. The study group required a lower dose of analgesics than the control group (*P* < 0.05) (Table 3).

### 3.3. Quality of Life

Before intervention, the two groups had similar SF-36 scores (*P* > 0.05). After intervention, the study group showed significantly higher scores of social functioning, role emotional, mental health, role physical, and bodily pain (84.02 ± 9.88, 83.29 ± 10.03, 81.94 ± 10.46, 83.82 ± 11.61, and 82.48 ± 10.14) than the control group (73.41 ± 13.54, 70.46 ± 11.52, 72.17 ± 10.45, 73.99 ± 14.51, and 71.98 ± 12.12) (*P* < 0.05) (Table 4).

### 3.4. Psychological Status

Before intervention, the two groups showed comparable psychological status scores (*P* > 0.05). After treatment, the study group had significantly lower SAS and SDS scores (46.08 ± 4.45 and 49.95 ± 4.02) than the control group (55.21 ± 5.89 and 58.48 ± 6.17) (*P* < 0.05) (Table 5).

### 3.5. Complications

In the study group, there were 3 cases of infections, 1 case of pressure sores, and 1 case of oral ulcers, with an incidence of 7.35% (5/68). In the control group, there were 8 cases of infections, 6 case of pressure sores, 4 cases of oral ulcers, and 3 cases of venous thrombosis with an incidence of 30.88% (21/68). The study group was associated with a significantly lower incidence of complications than the control group (Table 6).

### 3.6. Satisfaction Rate

In the study group, there were 32 cases of highly satisfied, 33 cases of satisfied, 2 cases of less satisfied, and 1 case of dissatisfied, with a satisfaction rate of 98.53% (67/68). In the control group, there were 18 cases of highly satisfied, 24 cases of satisfied, 19 cases of less satisfied, and 7 cases of dissatisfied, with a satisfaction rate of 89.71% (61/68). The study group showed a higher satisfaction rate than the control group (*P* < 0.05) (Table 7).

## 4. Discussion

Pancreatic cancer is a common malignant tumor of the gastrointestinal tract, with the clinical characteristics of short disease duration and rapid progression. Currently, pancreatectomy is a well-recognized radical treatment, but it is associated with a high perioperative mortality rate of patients [15]. It has been demonstrated that the amelioration of poor surgical outcomes could be achieved by incorporating effective care after pancreatic cancer surgery, but specific care methods are still inconclusive. Comfort care is an effective nursing modality that maintains the continuity and coordination of care and assists patients to master self-care skills, which boosts recovery and improves the quality of life of patients [16]. Taemin et al. stated that comfort care contributes to improving clinical outcomes and reducing the incidence of postoperative complications in pancreatic cancer patients. Accordingly, this study aims to analyze the effects of comfort care on the quality of life, psychological status, and nursing satisfaction of postoperative pancreatic cancer patients to provide a reference for clinical practice [17].

The causes of postoperative pain in pancreatic cancer include psychological factors, misplaced drains, environmental influences, postoperative infection, and mechanical irritation. In the present study, patients receiving comfort care showed a significantly lower VAS score and required a lower dose of analgesics than those receiving conventional care, indicating milder pain in patients given comfort care than those receiving conventional care. The reason may be that comfort care effectively relieves the pain of patients through disease education, psychological guidance, and medication instruction and helps patients have a correct understanding of their condition, thereby reducing the use of analgesics [18]. Moreover, comfort care herein resulted in a better quality of life for patients and lower SAS and SDS scores than conventional care, indicating better quality of life benefits and psychological status management of patients. Conventional care focuses more on disease care yet overlooks the physiological and psychological needs of patients. By contrast, comfort care is a new patient-centered nursing model that satisfies patients' physical and psychological needs in addition to the treatment of disease. Therefore, the quality of life and psychological status of patients were better after comfort care than after conventional care, which is similar to the findings of Nikio et al. [19]. Besides, comfort care was associated with a significantly lower incidence of complications and a higher nursing satisfaction than conventional care. The reason may be that comfort care requires a three-step approach to drug relief for patients with postoperative pain and enhanced skin and oral care, thereby effectively preventing complications such as oral ulcers and pressure sores [20]. Furthermore, comfort care provides environmental care, meticulous psychological care, social comfort care, and physical comfort care to efficiently alleviate patients' pain and improve their quality of life and psychological status, resulting in fewer complications and high patient satisfaction.

## 5. Conclusion

Comfort care effectively alleviates the pain of patients after pancreatectomy, reduces the incidence of complications, and improves their quality of life, psychological status, and satisfaction, so it shows good potential for clinical application.

## Figures and Tables

**Table 1:** Comparison of baseline patient profile.

	Control group (*n* = 68)	Study group (*n* = 68)	*t*/*χ*^2^	*P*
Gender (male/female)	30/38	39/29	2.383	0.123
Age ($\bar{x}\pm s$, years)	43.03 ± 4.87	42.65 ± 5.61	0.422	0.674
Disease duration	2.23 ± 0.71	2.37 ± 0.58	1.259	0.210
Pathological type			0.127	0.938
HDA	24	26		
MDA	25	24		
DCC	19	18		

*Note.* HDA = highly differentiated adenocarcinoma, MDA = moderately differentiated adenocarcinoma, DCC = ductal cell carcinoma.

**Table 2:** Comparison of VAS scores ($\bar{x}\pm s$).

	Before intervention	After intervention
Control group (*n* = 68)	8.03 ± 1.11	6.51 ± 0.98
Study group (*n* = 68)	7.98 ± 1.32	2.17 ± 0.31
*t*	0.239	34.818
*P*	0.811	<0.001

**Table 3:** Comparison of analgesic use (*n*, (%)).

	Dezocine	Flurbiprofen axetil	No medicine	Medication rate
Control group (*n* = 68)	23	25	20	48 (70.59)
Study group (*n* = 68)	11	13	44	24 (35.29)
*χ* ^2^				17.00
*P*				<0.001

**Table 4:** Comparison of SF-36 scores ($\bar{x}\pm s$).

		Control group (*n* = 68)	Study group (*n* = 68)	*t*	*P*
Social functioning	Before	62.87 ± 10.85	63.01 ± 10.64	0.076	0.940
	After	73.41 ± 13.54^*∗*^	84.02 ± 9.88^*∗*^	5.220	<0.001

Role emotional	Before	65.65 ± 12.37	65.54 ± 11.17	0.054	0.957
	After	70.46 ± 11.52^*∗*^	83.29 ± 10.03^*∗*^	6.926	<0.001

Mental health	Before	63.45 ± 12.03	63.78 ± 11.72	0.162	0.872
	After	72.17 ± 10.45^*∗*^	81.94 ± 10.46^*∗*^	5.449	<0.001

Role physical	Before	62.41 ± 10.95	62.74 ± 10.21	0.182	0.856
	After	73.99 ± 14.51^*∗*^	83.82 ± 11.61^*∗*^	4.362	<0.001

Bodily pain	Before	62.87 ± 10.33	62.62 ± 10.34	0.141	0.888
	After	71.98 ± 12.12^*∗*^	82.48 ± 10.14^*∗*^	5.479	<0.001

*Note.* ^*∗*^indicates *P* < 0.05 between before and after intervention in the same group.

**Table 5:** Comparison of SAS and SDS scores ($\bar{x}\pm s$).

	SAS scores		SDS scores	
	Before	After	Before	After
Control group (*n* = 68)	65.84 ± 8.23	55.21 ± 5.89	64.98 ± 6.78	58.48 ± 6.17
Study group (*n* = 68)	66.07 ± 7.88	46.08 ± 4.45	64.73 ± 6.98	49.95 ± 4.02
*t*	0.166	10.199	0.212	9.552
*P*	0.868	<0.001	0.832	<0.001

**Table 6:** Comparison of complications (*n*, (%)).

	Infections	Pressure sores	Oral ulcers	Venous thrombosis	Incidence
Control group (*n* = 68)	8	6	4	3	21 (30.88)
Study group (*n* = 68)	3	1	1	0	5 (7.35)
*χ* ^2^	12.173				
*P*	<0.001				

**Table 7:** Comparison of satisfaction (*n*, (%)).

	Highly satisfied	Satisfied	Not very satisfied	Dissatisfied	Satisfaction rate
Control group (*n* = 68)	18	24	19	7	61 (89.71)
Study group (*n* = 68)	32	33	2	1	67 (98.53)
*χ* ^2^					4.781
*P*					0.029

## Data Availability

All data generated or analyzed during this study are included in this published article.

## References

[1] Torphy R. J., Fujiwara Y., Schulick R. D. (2020). Pancreatic cancer treatment: better, but a long way to go. *Surgery Today*.

[2] Neoptolemos J. P., Kleeff J., Michl P., Costello E., Greenhalf W., Palmer D. H. (2018). Therapeutic developments in pancreatic cancer: current and future perspectives. *Nature Reviews Gastroenterology & Hepatology*.

[3] Tempero M. A. (2019). NCCN guidelines updates: pancreatic cancer. *Journal of the National Comprehensive Cancer Network*.

[4] Ansari D., Tingstedt B., Andersson B. (2016). Pancreatic cancer: yesterday, today and tomorrow. *Future Oncology*.

[5] Ilic M., Ilic I. (2016). Epidemiology of pancreatic cancer. *World Journal of Gastroenterology*.

[6] Goral V. (2015). Pancreatic cancer: pathogenesis and diagnosis. *Asian Pacific Journal of Cancer Prevention*.

[7] Chabanolle C. (2018). Preventive approach of oral care for comfort and hydration. *Revue de l’Infirmiere*.

[8] Stephens E. H., Dearani J. A., Qureshi M. Y. (2021). Toward eliminating perinatal comfort care for prenatally diagnosed severe congenital heart defects: a vision. *Mayo Clinic Proceedings*.

[9] Yates P. (2017). Symptom management and palliative care for patients with cancer. *Nursing Clinics of North America*.

[10] Yabrodi M., Mastropietro C. W. (2017). Hypoplastic left heart syndrome: from comfort care to long-term survival. *Pediatric Research*.

[11] Vincent J. L., Shehabi Y., Walsh T. S. (2016). Comfort and patient-centred care without excessive sedation: the eCASH concept. *Intensive Care Medicine*.

[12] Sung Y. T., Wu J. S. (2018). The visual analogue scale for rating, ranking and paired-comparison (VAS-RRP): a new technique for psychological measurement. *Behavior Research Methods*.

[13] Takeuchi E., Kim Y., Shaffer K. M., Cannady R. S., Carver C. S. (2020). Fear of cancer recurrence promotes cancer screening behaviors among family caregivers of cancer survivors. *Cancer*.

[14] Dunstan D. A., Scott N., Todd A. K. (2017). Screening for anxiety and depression: reassessing the utility of the Zung scales. *BMC Psychiatry*.

[15] Wittenberg E., Reb A., Kanter E. (2018). Communicating with patients and families around difficult topics in cancer care using the COMFORT communication curriculum. *Seminars in Oncology Nursing*.

[16] Kasvis P., Kilgour R. D. (2021). Diet and exercise interventions in patients with pancreatic cancer: a scoping review. *Pancreas*.

[17] Lee E. J., Seo S. Y., Kim I. H. (2020). Effect of cancer awareness on treatment decision for pancreatic cancer patients. *Korean Journal of Gastroenterology*.

[18] Partelli S., Sclafani F., Barbu S. T. (2021). European cancer organisation essential requirements for quality cancer care (ERQCC): pancreatic cancer. *Cancer Treatment Reviews*.

[19] Lei Y., Liu B., Su L. (2020). Perioperative nursing of patients with pancreatic cancer treated with a nanoknife. *Journal of Nanoscience and Nanotechnology*.

[20] Eaton L. H., Brant J., McLeod K., Hsing Yeh C. (2017). Nonpharmacologic pain interventions: a review of evidence-based practices for reducing chronic cancer pain. *Clinical Journal of Oncology Nursing*.

